# Revisiting the Effect of Anthropomorphizing a Social Cause Campaign

**DOI:** 10.1371/journal.pone.0138886

**Published:** 2015-09-25

**Authors:** Lisa A. Williams, Barbara Masser, Jessie Sun

**Affiliations:** 1 School of Psychology, UNSW Australia, Sydney, NSW, Australia; 2 School of Psychology, The University of Queensland, St Lucia, QLD, Australia; 3 Melbourne School of Psychological Sciences, University of Melbourne, Parkville, VIC, Australia; Tilburg University, NETHERLANDS

## Abstract

Recent research suggests that anthropomorphism can be harnessed as a tool to boost intentions to comply with social cause campaigns. Drawing on the human tendency to extend moral concern to entities portrayed as humanlike, it has been argued that adding personified features to a social campaign elevates anticipated guilt at failing to comply, and this subsequently boosts intentions to comply with that campaign. The present research aimed to extend extant research by disentangling the effects of emotional and non-emotional anthropomorphism, and differentiating amongst other emotional mechanisms of the anthropomorphism-compliance effect (namely, anticipated pride and anticipated regret). Experiment 1 (*N* = 294) compared the effectiveness of positive, negative, and emotionally-neutral anthropomorphized campaign posters for boosting campaign compliance intentions against non-anthropomorphized posters. We also measured potential mechanisms including anticipated guilt, regret, and pride. Results failed to support the anthropomorphism-compliance effect, and no changes in anticipated emotion according to anthropomorphism emerged. Experiments 2 (*N* = 150) and 3 (*N* = 196) represented further tests of the anthropomorphism-compliance effect. Despite high statistical power and efforts to closely replicate the conditions under which the anthropomorphism-compliance effect had been previously observed, no differences in compliance intention or anticipated emotion according to anthropomorphism emerged. A meta-analysis of the effects of anthropomorphism on compliance and anticipated emotion across the three experiments revealed effect size estimates that did not differ significantly from zero. The results of these three experiments suggest that the anthropomorphism-compliance effect is fragile and perhaps subject to contextual and idiographic influences. Thus, this research provides important insight and impetus for future research on the applied and theoretical utility of anthropomorphizing social cause campaigns.

## Introduction

Recently, Ahn, Kim, and Aggarwal proposed a novel way of increasing compliance with a social campaign, namely by anthropomorphizing a campaign poster [1]. Drawing on the broader literature on anthropomorphism (see [2] for a review), Ahn and colleagues reasoned that the presence of humanlike characteristics may increase moral concern, and thereby boost intentions to support a social cause. That is, a social cause that is portrayed in a humanlike manner will elicit the same regard as is extended towards other humans, including the desire to protect it from harm [2]. Insofar as the campaign is aimed towards increasing compliance with protective behaviors, anthropomorphizing the campaign should boost campaign compliance.

Across three experiments, Ahn et al. demonstrated support for their proposed anthropomorphism-compliance effect: individuals exposed to an anthropomorphized social campaign were more willing to comply with the campaign, compared with individuals exposed to the same environmental campaign presented without anthropomorphized features [1]. Specifically, in Ahn et al.’s Study 1, participants viewed either an anthropomorphized energy conservation poster, which featured a light bulb with humanlike facial features and a personified message, or a non-anthropomorphized version, in which the light bulb lacked facial features and was accompanied by an abstract message. In line with the hypothesized anthropomorphism-compliance effect, participants who viewed the anthropomorphized poster reported higher intention to comply with the campaign. Additional studies replicated the anthropomorphism-compliance effect in the context of a food waste recycling campaign (Study 2) and a tree-planting campaign (Study 3). Ahn et al.’s Study 3 also comprised a field study, demonstrating the effects of anthropomorphism on the number of donors and amount of money donated to the campaign. The basic anthropomorphism-compliance effect has been observed by another research group using visual and verbal anthropomorphized stimuli [3].

Ahn and colleagues reasoned that people might comply with an anthropomorphized social campaign because they wish to avoid feeling guilty about causing harm to the anthropomorphized entity through non-compliance. Extant theory and empirical evidence support this premise. Guilt is an aversive emotional state experienced when one feels responsible for harming another person through their actions or inactions [4]. When anticipating guilt, individuals modify their intentions and behaviors to avoid experiencing this aversive state [5–15]. The positive behavioral effects of anticipated guilt are frequently observed in the context of environmental behavior [16–18]. Consistent with this, Ahn and colleagues (Study 2) demonstrated that participants who viewed an anthropomorphized poster advocating an environmentally-friendly behavior anticipated higher levels of guilt for not complying with the campaign, compared with participants who viewed a non-anthropomorphized poster. Further, elevated levels of anticipated guilt mediated the boost in intention to comply with the anthropomorphized campaign.

Given the potential cost-effectiveness and utility of anthropomorphizing social causes, this phenomenon merits further investigation. We identified two promising directions for extending Ahn and colleagues’ original findings: (1) disentangling emotional from non-emotional anthropomorphism, and (2) identifying and differentiating amongst other emotional mechanisms of the anthropomorphism-compliance effect.

Anthropomorphism is not a unitary construct [19]; extension of emotional capacities to nonhuman entities is but one aspect of the process, which can include the attribution of other experiential (e.g., hunger, personality, consciousness) and agentic (e.g., self-control, morality, thought) mental capacities [20]. The stimuli used by Ahn and colleagues, however, strongly emphasized emotional anthropomorphism. That is, their stimuli included anthropomorphic cues that also conveyed emotional capacity, and specifically, negative emotional capacity. The anthropomorphized light bulb from Study 1 and the anthropomorphized recycling bin from Study 2 both featured facial expressions characteristic of sadness (i.e., a tear drop and a frowning mouth [21,22]), whereas the wide eyes of the tree in Study 3’s anthropomorphized poster are characteristic of a fearful or surprised state [21,23]. Given this, it is not clear whether emotional cues are required for the anthropomorphism-compliance effect. Recent findings suggest that verbal person-based references to nature (e.g., Mr., him) are sufficient to elevate intentions to engage in environmentally-friendly behaviors [3]. No research to date, however, has directly compared the relative effectiveness of emotional- and non-emotional anthropomorphic cues on boosting campaign compliance intention nor considered whether the valence of emotional cues (e.g., smiling vs. frowning) may further moderate the effects.

With regard to mechanisms, it is plausible that anticipated pride also serves as a mechanism for the anthropomorphism-compliance effect. In many ways, pride represents the positive emotional counterpart to guilt. Guilt arises from violating or failing to meet moral standards, whereas pride emerges through meeting or exceeding those standards [15,24,25]. Just as anticipated guilt can shape behavior, so too can anticipated pride [26,27]. Put succinctly, individuals are motivated to engage in actions that they anticipate will bring about the experience of pride. Such effects are observed in the context of environmental and social causes: anticipated pride boosts intention to engage in environmentally-friendly actions [28] and purchase fair trade products [9] over and above the motivating effects of anticipated guilt.

In a related vein, we saw theoretical value in differentiating the effects of anticipated guilt from a related, yet theoretically distinct state: anticipated regret. Both guilt and regret are negative emotional states that can result from either action or inaction. These two emotions also function in a similar way; both anticipated regret and guilt serve to encourage behaviors (or restraint from behaviors) so as to preclude or attenuate the future experience of that emotion. Theories regarding anticipated regret highlight this emotion’s relevance in decision-making contexts that involve uncertainty about future outcomes [29–31]. Regret arises when a person engages in counterfactual thinking and concludes that their current experience would be better had they made a different choice [32,33]. For this reason, most research on anticipated regret has focused on domains of choice that directly impact the self (e.g., exercise, condom use, playing the lottery, health screening; see [34–36] for meta-analytic and literature reviews), though many studies have examined anticipated regret in more social contexts (e.g., organ and blood donation, interpersonal decision-making games [31,37–41]). Of most relevance to the current research, anticipated regret is highly predictive of environmental behavioral intentions and action [42].

Despite the relevance of regret in deciding to comply with social causes such as environmental action, it may be less pertinent than anticipated guilt in the context of an *anthropomorphized* social campaign. As mentioned above, anthropomorphism arouses moral concern relating to the prospect of harming another entity—a form of interpersonal harm. Zeelenberg and Breugelman have suggested that guilt is predominantly felt in situations of interpersonal harm, whereas regret is felt in both interpersonal and intrapersonal situations of harm [43]. Moreover, when paired with the predominance of counterfactual thinking in the elicitation of regret [32,33], it is plausible that, unless prompted to think about the future, an anthropomorphized campaign may not elicit anticipated regret to the same degree as anticipated guilt. This possibility remains an empirical question, as no studies to date have directly compared the relevance of anticipated guilt and regret in the context of social campaigns more generally, or anthropomorphized social campaigns specifically.

The two extensions we undertook (i.e., manipulating emotional anthropomorphizing cues and measuring multiple potential emotional mediators) enabled us to test an exploratory question: whether anticipated pride, guilt, and regret would be differentially evoked depending on the nature of the emotional anthropomorphizing cues. Specifically, since frowning conveys disapproval and cues potential transgression [44–46], anthropomorphizing a campaign with a sad expression may be more likely to evoke anticipated guilt and regret and less likely to evoke anticipated pride. Likewise, as smiling conveys praise or approval [44,46–49] and praise is a key component in the elicitation of pride [50–52], anthropomorphizing a campaign with a happy expression may produce greater levels of anticipated pride.

Across three experiments, we examined the effect of anthropomorphizing an energy conservation campaign on intention to comply with that campaign. Further, we tested the roles of anticipated guilt, regret, and pride as mechanisms of the anthropomorphism-compliance effect. In Experiment 1, we aimed to replicate and extend the findings of Ahn et al. (Study 1) by including two additional anthropomorphized posters, which portrayed happy and neutral expressions. To replicate and extend tests of the mechanism underlying the anthropomorphism-compliance effect, we included measures of anticipated regret and pride as well as of anticipated guilt. Experiments 2 and 3 represented attempts to replicate the anthropomorphism-compliance effect with just the sad-anthropomorphized and non-anthropomorphized posters, given that we failed to observe significant differences in intention as a function of anthropomorphism condition in Experiment 1.

In all three experiments, we expected to replicate Ahn et al.’s core findings. Specifically, we hypothesized that participants who viewed a sad-anthropomorphized poster, compared to a non-anthropomorphized poster, would report higher levels of intention to comply with the energy conservation campaign. We also expected that anthropomorphism would elicit higher levels of anticipated guilt for non-compliance, and that anticipated guilt would mediate the effect of anthropomorphism on intention.

We further predicted that emotional anthropomorphism might represent a particularly strong case of the anthropomorphism-compliance effect. Since emotionally-framed and emotionally-evocative appeals—within limits—have been demonstrated to positively influence environmental attitudes and behavior [53–55], it stands to reason that posters that contain emotional cues should induce campaign compliance more effectively than posters without those cues. Therefore, we anticipated that happy-anthropomorphism would be as effective as sad-anthropomorphism, with both being more effective than neutral-anthropomorphism.

With regard to emotional mechanisms, we expected anticipated pride to serve as an additional, concurrent mechanism via which anthropomorphism boosts social cause compliance. Further, we predicted that social cause anthropomorphism would evoke higher levels of anticipated guilt than anticipated regret, given that anthropomorphism arouses moral concern for interpersonal harm and that guilt is more specifically aligned with interpersonal harm than is regret.

In summary, across three experiments, we aimed to replicate and extend Ahn and colleagues’ findings regarding the effect of anthropomorphizing a social cause campaign on intentions to comply with that campaign. Although we failed to replicate the anthropomorphism-compliance effect, the findings of these three experiments represent an important contribution to the literature on anthropomorphism as a tool for boosting social cause campaign compliance, by pointing to the need to investigate boundary conditions of this effect. In doing so, our findings also hold important applied implications for governments, companies, and other bodies that may consider deploying anthropomorphism in hope of increasing social cause compliance. In short, our results suggest that the anthropomorphism-compliance phenomenon is not yet sufficiently understood to be effectively applied.

In this manuscript, we report all measures, conditions, and data exclusions, as well as our rules for determining sample sizes in each experiment. Data files for Experiments 1–3 can be accessed at https://osf.io/b5z84/.

## Experiment 1

Experiment 1 represented an attempt to extend Ahn and colleagues’ findings in two ways: (1) disentangle emotional from non-emotional anthropomorphism, and (2) differentiate amongst other emotional mechanisms of the anthropomorphism-compliance effect. To distinguish between the effects of anthropomorphism per se and expressed emotion, we included neutral-anthropomorphized and happy-anthropomorphized versions of the campaign poster, in addition to the non-anthropomorphized and sad-anthropomorphized posters used in Ahn et al.’s original Study 1. Further, we measured anticipated guilt, pride, and regret as potential mechanisms of the anthropomorphism-compliance effect.

### Method

#### Participants and design

Participants (*N* = 301) recruited from Amazon’s Mechanical Turk completed the experiment in exchange for monetary compensation. We conducted a required sample size analysis using G*Power [56] for the effect size observed by Ahn et al., *d* = 0.45, an alpha level of .05, and a power level of .80. This analysis indicated a required sample size of 62 per condition. In anticipation of exclusions due to inattention, we decided to collect a minimum of 75 participants per condition in Experiments 1 and 2.

The sample comprised 101 females and 200 males (*M*_age_ = 29.98, *SD*_age_ = 10.39) who self-reported as White/Caucasian (*n* = 217), Asian (*n* = 35), African-American (*n* = 22), Hispanic (*n* = 17), Native American (*n* = 3), Arab/Middle Eastern (*n* = 1), or Other (*n* = 6). Data from seven participants were excluded because they failed to answer correctly an item included to screen for attention during the survey, resulting in an analyzed sample of 294 participants.

Participants were randomly assigned to one of eight conditions, which included four conditions that adopted a different campaign (i.e., blood donation). and are reported in a supplement available at https://osf.io/b5z84/. Deployment of this set of conditions was driven by exploratory interest in the possible extension of the anthropomorphism-compliance effect into non-environmental contexts. The four conditions were analogous to those reported here: neutral-anthropomorphism, sad-anthropomorphism, neutral-anthropomorphism, and happy-anthropomorphism. Stimuli and a comprehensive write-up of the method and results from these four conditions are accessible on the OSF site for this manuscript (https://osf.io/b5z84/).

Of the four relevant conditions, two conditions utilized the same posters as Ahn et al.’s Study 1, thus comprising the non-anthropomorphism (*n* = 72) and sad-anthropomorphism (*n* = 73) conditions. Two new conditions were created: neutral-anthropomorphism (*n* = 75) and happy-anthropomorphism (*n* = 74).

The protocol for this experiment, including the procedure for providing informed consent, was approved by the University of New South Wales Human Research Ethics Approval Panel C (Approval #133–178). All participants provided informed consent for participation in the protocol by entering their initials into a textbox, which was recorded by the survey software. As per the Conditions of Use of Mechanical Turk, all registered users are required to be at least 18 years of age. As such, specific procedures for obtaining the consent of minors were not required.

#### Procedure

**Fig 1:**
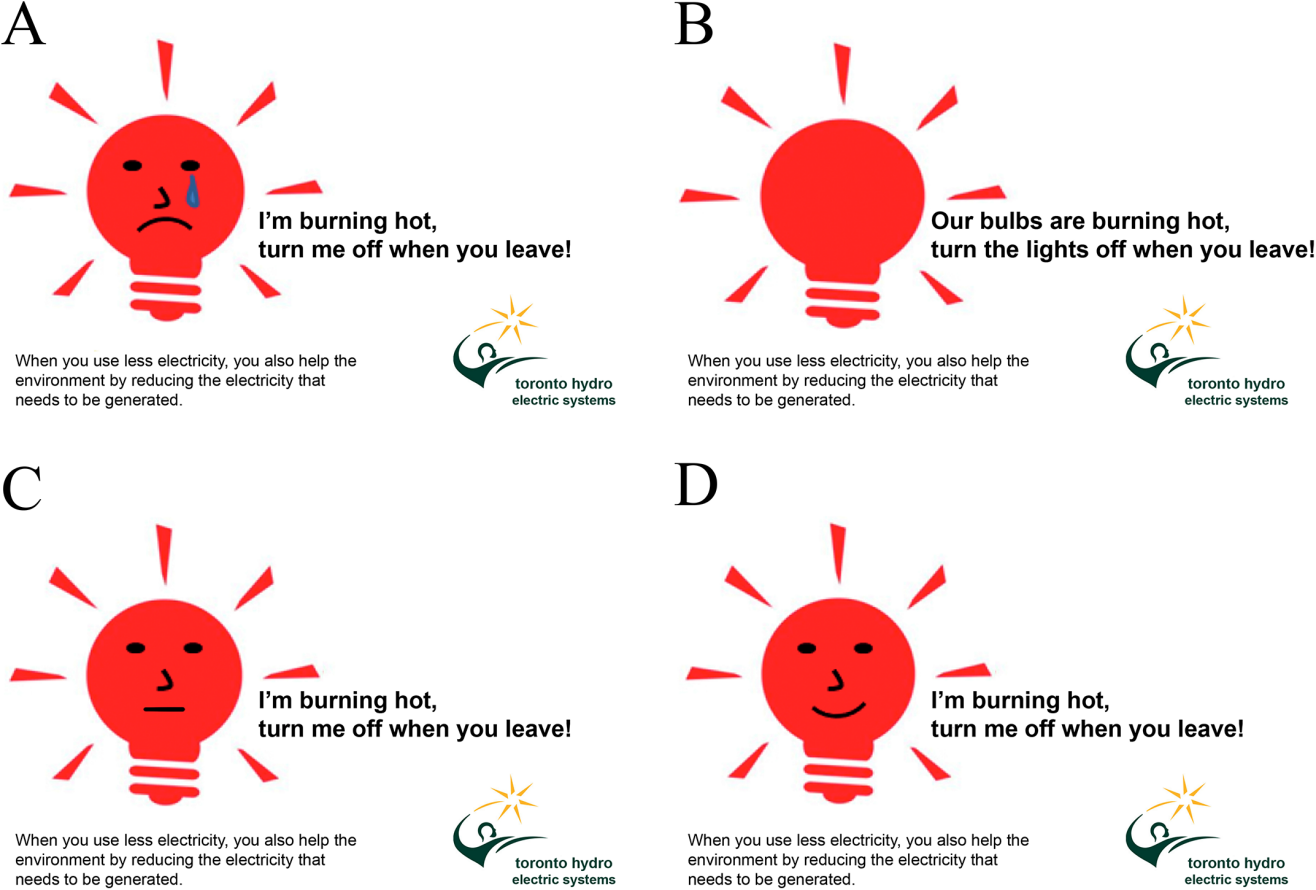
Campaign poster stimuli used in Experiment 1. The four campaign posters were presented to participants on a between-subjects basis. The sad-anthropomorphism (Panel A) and non-anthropomorphism (Panel B) posters replicate the stimuli utilized in Ahn et al.’s Study 1. The neutral-anthropomorphism (Panel C) and happy-anthropomorphism (Panel D) posters were created for the purpose of Experiment 1 by removing the teardrop and changing the mouth to a flat line (neutral) or inverting the frown (happy).

Participants were told that they would be evaluating a new community campaign poster and were randomly assigned to view one of four energy conservation campaign posters. In the three anthropomorphism conditions, participants viewed a version of the poster that featured a light bulb with humanlike features (with eyes, a nose and a mouth), with either a sad, neutral, or happy expression. The sad poster was the same as that used in Ahn et al.’s Study 1. The happy and neutral posters were created by removing the teardrop and either inverting the frown (happy poster) or replacing the frown with a straight line (neutral poster). The three anthropomorphized posters also contained a message stating, “I’m burning hot, turn the lights off when you leave!,” as per the original stimuli. In the non-anthropomorphism condition, participants saw a version of the poster that featured a light bulb with no humanlike features, and the message, “Our bulbs are burning hot, turn the lights off when you leave!” as per the original stimuli. Poster images appear in Fig 1.

After viewing the poster for at least 8 s, participants completed measures assessing anticipated emotions and intentions to comply with the campaign. After completing these measures, and before being debriefed, participants responded to demographic questions including questions assessing age, ethnicity and gender.

#### Measures

Imagining that the campaign was launched in their local area, participants indicated their anticipated guilt and regret as a result of not conserving energy (“To what extent would you feel … if you did not conserve energy?”). Anticipated guilt was assessed through responses to “ashamed”, “irresponsible”, and “guilty” (adapted from Ahn et al., Study 2) and anticipated regret through responses to “regretful” and “remorseful”. The question stem for anticipated pride referred to conserving (rather than not conserving) energy and used the descriptors “proud” and “accomplished”. Items were averaged to form scales of anticipated guilt (α = .88), anticipated regret (α = .90) and anticipated pride (α = .85). Responses were made on 8-point scales anchored by *not at all* and *extremely*.

Participants indicated their intention to conserve energy if the campaign were implemented in their local area. Specifically, participants rated the following items: “How likely is it that you will participate in energy conservation?,” *very unlikely*–*very likely*; “How likely would you be to conserve energy?,” *very unlikely*–*very likely*; “I will conserve energy,” *strongly disagree*–*strongly agree*. These items were averaged to form an index of campaign compliance intention (α = .95). The first of these items was one of the compliance items utilized by Ahn and colleagues. All items were rated on 9-point scales. The attention-check question (“When you get to this item, please click the second option from the left”) was embedded within this measure.

### Results and discussion

Descriptive statistics for measured constructs appear in Table 1 and effect size estimates and corresponding 95% confidence intervals based on the noncentral *t* distribution [57], using guidelines outlined by Wuensch [58], appear in Table 2. A series of planned comparisons were conducted to compare the non-anthropomorphism condition with each of the three anthropomorphism conditions with regard to intention. In contrast to Ahn et al.’s Study 1, intention to conserve energy, as measured via the 3-item index, did not differ between participants who viewed the non-anthropomorphized or sad-anthropomorphized posters, *t*(290) = 1.28, *p* = .20, between participants who viewed the non-anthropomorphized or neutral-anthropomorphized poster, *t*(290) = 0.01, *p* = .995, or between participants who viewed the non-anthropomorphized or happy-anthropomorphized poster, *t*(290) = 0.88, *p* = .38. Analysis of the single intention item used by Ahn et al. revealed a similar pattern, *t*s < 1.03, *p*s > .30. We thus failed to replicate the effect of anthropomorphism on campaign compliance intention across any of the anthropomorphism conditions.

**Table 1:** Descriptive statistics for measured constructs in Experiments 1, 2 and 3.

		Compliance Intention (1-item)	Compliance Intention (3-item)	Campaign Evaluation (5-item)	Anticipated Guilt	Anticipated Regret	Anticipated Pride
Experiment 1							
	Non-anthropomorphism	6.11 (2.05)	6.06 (1.99)	-	4.38 (1.54)	4.07 (1.68)	5.33 (1.73)
	Sad-anthropomorphism	6.44 (2.00)	6.45 (1.83)	-	4.28 (1.68)	4.10 (1.76)	5.36 (1.73)
	Neutral-anthropomorphism	6.09 (1.78)	6.06 (1.75)	-	4.17 (1.73)	3.98 (1.74)	5.37 (1.63)
	Happy-anthropomorphism	6.38 (1.81)	6.32 (1.71)	-	4.61 (1.72)	4.61 (1.70)	5.63 (1.53)
Experiment 2							
	Non-anthropomorphism	6.38 (2.00)	6.35 (2.05)	-	4.57 (1.91)	4.33 (2.01)	5.59 (1.78)
	Sad-anthropomorphism	5.96 (2.01)	6.06 (1.92)	-	4.81 (1.78)	4.67 (1.76)	5.12 (1.79)
Experiment 3							
	Non-anthropomorphism	5.48 (2.03)	-	5.02 (1.66)	5.00 (1.85)	4.74 (1.92)	5.76 (1.79)
	Sad-anthropomorphism	5.34 (2.13)	-	4.84 (1.82)	5.25 (1.72)	4.85 (1.98)	5.69 (1.95)

*Note*. Standard deviation values appear in parentheses next to mean values.

**Table 2:** Estimate and confidence intervals around effect size *d*s of the comparison between non-anthropomorphism and sad-anthropomorphism in Experiments 1, 2, and 3.

	Ahn et al.	Experiment 1	Experiment 2	Experiment 3	Meta-analyzed Effect Size
Compliance Intention (1-item)	-	0.16 [-0.16, 0.49]	-0.21 [-0.53, 0.11]	-0.07 [-0.35, 0.21]	-0.04 [-0.22, 0.13]
Compliance Intention (3-item)	-	0.20 [-0.12, 0.53]	-0.15 [-0.47, 0.17]	-	-
Campaign Evaluation (5-item)	0.45	-	-	-0.10 [-0.38, 0.18]	-
Anticipated Guilt	0.44	-0.06 [-0.38, 0.27]	0.13 [-0.19, 0.45]	0.14 [-0.14, 0.42]	0.08 [-0.10, 0.25]
Anticipated Regret	-	0.02 [-0.31, 0.34]	0.18 [-0.14, 0.50]	0.06 [-0.22, 0.34]	0.08 [-0.09, 0.26]
Anticipated Pride	-	0.02 [-0.30, 0.35]	-0.26 [-0.58, 0.06]	-0.04 [-0.32, 0.24]	-0.09 [-0.27, 0.09]
Anthropomorphism Check	-	-	-	0.92 [0.62, 1.21]	-
Current Mood	-	-	-	-0.07 [-0.35, 0.21]	-

*Note*. Effect sizes from Ahn et al. are drawn from Study 1 for campaign evaluation and Study 2 for anticipated guilt. Positive effects reflect comparisons in which the sad-anthropomorphism mean was higher than the non-anthropomorphism mean. Experiment 1–3 confidence interval values, which appear in brackets, represent 95% confidence intervals based on the noncentral *t* distribution [57], using guidelines outlined by Wuensch [58]. Note also that effect sizes and confidence intervals for Experiment 1 were calculated from between-condition independent-sample *t*-tests in light of the requirement for corresponding degrees of freedom requirements for noncentral *t* confidence interval estimation. Meta-analyzed effect sizes and respective 95% confidence intervals are based on Experiments 1–3 (see Meta-Analytic Summary of Experiments).

In contrast to Ahn et al.’s Study 2, levels of anticipated guilt did not differ according to the poster viewed, *t*s < .84, *p*s > .40. A similar pattern emerged for anticipated pride, *t*s < 1.10, *p*s > .27, and anticipated regret, *t*s < 1.92, *p*s > .06. Because none of the anthropomorphism conditions impacted levels of these potential mediators, tests of indirect effects were not appropriate.

We thus failed to observe an effect of any form of anthropomorphism on intention or anticipated guilt, pride or regret. However, Experiment 1 faced two limitations. First, given the wider geographic distribution of our online sample, the logo, which read “toronto hydro”, may have lacked the geographic relevance achieved by Ahn et al.’s Study 1. Second, since the poster only appeared on a single screen of the survey, for a relatively short minimum viewing period of 8 s, it is possible that participants did not view the poster for long enough for an effect on intention or anticipated emotions to emerge. We therefore addressed these limitations in Experiment 2.

## Experiment 2

The aim of Experiment 2 was to attempt to replicate the anthropomorphism-compliance effect obtained by Ahn et al. while addressing two limitations of Experiment 1: the geographic relevance of the poster and the duration of exposure to the poster. We dropped the neutral- and happy-anthropomorphism conditions to focus on replicating the anthropomorphism-compliance effect with the original two conditions used by Ahn and colleagues.

### Method

Participants (*N* = 150) recruited from Amazon’s Mechanical Turk completed the experiment online for monetary compensation. The sample comprised 48 females and 102 males (*M*_age_ = 28.81, *SD*_age_ = 8.16) who self-reported as White/Caucasian (*n* = 115), Asian (*n* = 14), African-American (*n* = 6), Hispanic (*n* = 10), Native American (*n* = 1) or Other (*n* = 4). All participants correctly responded to the attention-screening question (i.e., “When you get to this item, please click the option that contains three words;” correct answer: *not at all*), and therefore no data were excluded from analysis.

All procedures and measures were the same as Experiment 1, with three exceptions. First, participants were only assigned to view the non-anthropomorphized (*n* = 73) or sad-anthropomorphized (*n* = 77) versions of the posters, following Ahn et al.’s Study 1. Second, the logo on the poster was changed from “toronto hydro electric systems” to “world hydro electric systems” with the goal of making the poster relevant to the wider geographic base of the sample. Third, after the initial 8 s minimum poster viewing, the posters also appeared at the top of the screens while participants completed the measures of anticipated emotions and intentions. This change was adopted to maximize exposure to the poster content. Confirming this, after excluding one participant for whom the survey timer did not record their progress, participants spent on average 73.96 s in total on the screens that presented the poster (*SD* = 31.26, range: 11.80–201.34). As in Experiment 1, all scales demonstrated high reliability: α_guilt_ = .93, α_regret_ = .92, α_pride_ = .93, α_3-item intention_ = .95.

The protocol for this experiment, including the procedure for providing informed consent, was approved by the University of New South Wales Human Research Ethics Approval Panel C (Approval #133–178). All participants provided informed consent for participation in the protocol by entering their initials into a textbox, which was recorded by the survey software. As per the Conditions of Use of Mechanical Turk, all registered users are required to be at least 18 years of age. As such, specific procedures for obtaining the consent of minors were not required.

### Results and discussion

Descriptive statistics for all measured constructs appear in Table 1 and effect size estimates and confidence intervals appear in Table 2. A series of independent-samples *t*-tests were conducted to compare levels of campaign compliance intention and anticipated emotion between the non-anthropomorphism and sad-anthropomorphism conditions. As in Experiment 1, participants who viewed the sad-anthropomorphized poster did not differ significantly from participants who viewed the non-anthropomorphized poster on intentions to conserve energy, measured either as a 3-item index or a single item, *t*s < 1.29, *p*s > .20. Further, participants in the two conditions did not differ in levels of anticipated guilt, pride, or regret, *t*s < 1.60, *p*s > .11.

Despite addressing the geographical relevance and viewing time limitations of Experiment 1, Experiment 2 also failed to replicate the anthropomorphism-compliance effect. We acknowledge that, in the attempt to address the issue of geographical relevance by representing a broad geographic area, this change may have inadvertently lost its local appeal by not being specific to a particular city. Experiments 1 and 2 also shared three key departures from the methodology reported by Ahn et al. (Study 1): the use of an online rather than laboratory sample, the omission of a manipulation and mood check, and the measurement of intention only instead of a broader measure of “campaign evaluation” that also included intention. Experiment 3 addressed these limitations.

## Experiment 3

Experiment 3 was conducted as a close replication attempt of Ahn et al.’s Study 1. The authors of the original research generously shared their materials, which we adapted in only two ways: we changed the city mentioned on the poster from Toronto to Brisbane (the city in which the research was conducted) and maintained the addition of anticipated guilt, pride and regret measures to enable the identification of potential process mechanisms. In order to align our sample and study characteristics with Ahn et al. as much as possible, participants in Experiment 3 were undergraduate students, who completed the experiment in a laboratory setting.

### Method

#### Participants and design

Participants (*N* = 196) were 134 female and 62 male (*M*_age_ = 21.32, *SD*_age_ = 5.36) undergraduate students at the University of Queensland in Australia. Given our orientation towards attempting to replicate Ahn et al., we conducted another required sample size analysis, raising the power level to .90, as per current recommendations for replication studies [59]. This analysis indicated a required sample size of 86 per condition. We set this as a minimum per-condition sample size for Experiment 3.

Participants completed the study in exchange for partial course credit. Participants were randomly allocated either to the non-anthropomorphism (*n* = 99) or the sad-anthropomorphism (*n* = 97) condition. This experiment did not include an attention check question and all collected data were included in the analyses reported below.

The protocol for this experiment, including the procedure for providing informed consent, was approved by the University of Queensland Behavioral and Social Sciences Ethical Review Committee (Approval #2014000243). All participants provided written informed consent for participation in the protocol by signing a consent form. In accordance with guidelines set in the Australian National Health and Mental Research Council’s National Statement on Ethical Conduct in Human Research, students enrolled in university courses are presumed to possess the capacity to provide consent. As such, specific procedures for obtaining the consent of minors were not required.

#### Procedure

Participants were told that they would be evaluating a new community campaign poster, and viewed either the non-anthropomorphized or sad-anthropomorphized poster. No time constraints were imposed during the poster viewing time. To maintain the local relevance of the poster used in the original research (i.e., “toronto hydro electric systems” with a sample from Toronto), we modified the poster so that the logo read “brisbane hydro electric systems”. All other aspects of the posters were exactly the same as the original stimuli. On the following pages of the paper questionnaire packet, participants rated their anticipated emotions, evaluations of the campaign, a manipulation check on anthropomorphism, and current mood, in this order. After completing these measures, and before being debriefed, participants indicated their age and gender.

#### Measures

Anticipated emotions were measured using the same items as in Experiments 1 and 2 (α_guilt_ = .85, α_regret_ = .90, α_pride_ = .83). In Experiment 3, we utilized the 5-item index used by Ahn et al. (Studies 1 and 2). This 5-item index included one item also used in Experiments 1 and 2 (“If the campaign were implemented in your local area, how likely would you be to participate in energy conservation?,” *very unlikely*–*very likely*). Participants also rated how much they liked the campaign and evaluated how favorable, effective, and successful the campaign would be. These five items were combined to form a 5-item campaign evaluation index (α = .93). All items were rated on 9-point scales.

As a manipulation check on anthropomorphism, participants rated the extent to which they saw the bulb in the poster as a person (1 = *Not at all person-like*, 9 = *Very person-like*) and the extent to which the bulb seemed alive (1 = *Not at all alive*, 9 = *Very alive*), as measured by Ahn et al. (author correspondence). These were combined into a 2-item index (α = .86). Participants also rated their current mood on four 17-point scales anchored from -8 to 8 with emotion terms used by Ahn and colleagues (*bad/good*, *disappointed/satisfied*, *sad/happy*, *displeased/pleased*). These were combined to form a single index of current mood (α = .94).

### Results and discussion

Effect size estimates and confidence intervals appear in Table 2. As evidence of the success of the anthropomorphism manipulation, participants who viewed the sad-anthropomorphized poster rated the bulb as significantly more person-like and alive (*M* = 4.63, *SD* = 2.44) than participants who viewed the non-anthropomorphized poster (*M* = 2.65, *SD* = 1.81), *t*(177) = 6.43, *p* < .001. Because Levene’s test indicated unequal variances, *F*(1,194) = 17.21, *p* < .001, we adjusted the degrees of freedom from 194 to 177 for this test. This confirms that participants were in fact attending to the content. Replicating Ahn et al., participants’ current mood did not differ according to condition (*M*_non-anthro_ = 3.02, *SD*_non-anthro_ = 3.05, *M*_sad-anthro_ = 2.82, *SD*_sad-anthro_ = 2.99), *t*(194) = 0.46, *p* = .65.

Descriptive statistics for intention, campaign evaluation, and anticipated emotion appear in Table 1. Consistent with Experiments 1 and 2, and thus failing to replicate Ahn et al.’s Studies 1 and 2, participants who viewed the sad-anthropomorphized poster did not differ from participants who viewed the non-anthropomorphized poster on intention (single-item), their evaluation of the campaign (5-item), or anticipated guilt, *t*s < 1.00, *p*s > .32. Nor did participants in the two conditions vary on anticipated pride or anticipated regret, *t*s < 0.39, *p*s > .70.

Even with this close replication attempt, we failed to observe an effect of anthropomorphism on intention or anticipated guilt. The only relevant methodological departures from Ahn and colleague’s Study 1 were the inclusion of measures of anticipated emotion and the use of an Australian rather than North American sample. We also note that the anticipated emotion measures we deployed differ slightly to those used in Ahn et al.’s Study 2; we used 8-point instead of 9-point scales and slightly different terms to measure guilt (*guilt*, *shame*, *irresponsible*, as compared to Ahn et al.’s *guilt*, *shame*, *responsibility*, *accountability*). However, these minor departures should not have nullified what appeared to be a robust effect on intention.

## Meta-Analytic Summary of Experiments

We conducted a meta-analysis of our three experiments to obtain more precise parameters for the overall effect of viewing a non-anthropomorphized or sad-anthropomorphized poster on campaign compliance intention. Meta-analytic approaches enable generalization beyond minor differences in protocol and sample demographics between specific experiments included in the meta-analysis.

A post-hoc power analysis using G*Power [56] revealed that, given the total meta-analytic sample of 491 participants (*n*_non-anthro_ = 244, *n*_sad-anthro_ = 247) and an alpha level of .05, we obtained a power level above .99 to detect Ahn et al.’s Study 1 effect size of *d* = 0.45. Meta-analytic calculations were deployed using SPSS syntax developed by Wilson [60]. Effect size input comprised Cohen’s *d* corresponding to the independent samples t-test comparing campaign compliance intention (single-item) in the sad-anthropomorphism and non-anthropomorphism conditions from each of the three experiments (see Table 2, row 1). These effect size estimates were then adjusted using a standard small sample size bias correction and weighted by inverse variance, as advocated by Lipsey and Wilson [61]. A *Q*-test for heterogeneity across effect sizes revealed no significant violations of homogeneity, *Q*(2) = 2.57, *p* = .28. As such, we utilized a fixed effects model [61], which yielded an effect size estimate that did not differ significantly from zero (-0.04, 95% CI [-0.22, 0.13], *p* = .63).

For anticipated guilt, we obtained a power level above .99 to detect Ahn et al.’s Study 2 effect size of *d* = 0.44. Given that we did not observe violations of homogeneity, *Q*(2) = 0.97, *p* = .62, we deployed a fixed effects model that yielded an effect size estimate that did not differ significantly from zero (0.08, 95% CI [-0.10, 0.25], *p* = .39).

Rounding out the meta-analysis, we observed effect size estimates for anticipated regret (0.08, 95% CI [-0.09, 0.26], *p* = .35) and anticipated pride (-0.09, 95% CI [-0.27, 0.09], *p* = .33) that did not differ significantly from zero. In both cases, fixed-effects models were used, given that homogeneity assumptions were met (regret: *Q*(2) = 0.51, *p* = .77; pride: *Q*(2) = 1.62, *p* = .45).

In sum, the meta-analysis revealed effect size estimates close to zero, with narrower 95% CIs around these estimates than were attainable in each of the individual experiments (see Table 2). Thus, we found no evidence that anthropomorphizing an environmental campaign poster using sad-anthropomorphic cues changed levels of intention to comply with the campaign, or anticipated emotion, across our three experiments.

## General Discussion

The present research aimed to replicate and extend recent findings that anthropomorphizing a social campaign increased compliance intentions [1,3], and that this relationship was mediated by an increase in anticipated guilt [1]. Following our inability to establish these key effects in Experiment 1 with the original sad-anthropomorphism or novel neutral- and happy-anthropomorphism conditions, we attempted to more closely replicate Ahn and colleagues’ Study 1 with the original conditions only (Experiments 2 and 3) and using the original materials and a comparable sample and setting (Experiment 3). Across three experiments, however, we found no significant differences in intention or anticipated guilt between participants who viewed a sad-anthropomorphized poster or a non-anthropomorphized poster. A meta-analysis of the effects observed in the three experiments revealed anthropomorphism-compliance effect size estimates (and anticipated emotion effect size estimates) that did not differ significantly from zero.

Each of our experiments had minor departures from the original studies, which we attempted to eliminate with each subsequent experiment. Indeed, Experiment 3 used the original materials and a comparable sample, and was conducted in a laboratory setting. As our closest replication attempt, Experiment 3 should therefore carry the greatest ‘informational weight’ in judging replicability; yet, despite these measures, we were unable to establish any significant effects on intention or anticipated guilt. The only minor departures in Experiment 3 from Ahn et al.’s Study 1 were changing the named city from Toronto to Brisbane on the poster to maintain its geographical relevance, the use of an Australian instead of a North American sample, and the additional measurement of anticipated emotions. However, we would expect that these small departures should not have nullified the anthropomorphism-compliance effect if it was substantial, robust, and had practical relevance.

Our failure to observe differential effects of positive, negative, neutral and non-anthropomorphized campaign posters on campaign compliance intention does not mean that anthropomorphic cues hold no potential for increasing compliance. For example, recent evidence suggests that the presence of eye-like stimuli can promote prosocial giving and environmentally-friendly behavior in field settings, mirroring the effect of anthropomorphism on donations that Ahn et al. found in Study 3 [62–66]. However, our findings do suggest that the effects of anthropomorphism on the effectiveness of social campaigns may be subject to a larger array of factors than originally thought, and that perhaps were unmeasured or not manipulated in Ahn and colleagues’ and our experiments.

In relation to the effects of anthropomorphism on campaign compliance, recent evidence suggests that individual difference variables moderate the anthropomorphism-compliance effect: a desire for effectance, or control over one’s life, and the need for social connection [67,68]. Specifically, anthropomorphic appeals resulted in a stronger intention to engage in conservation than non-anthropomorphic appeals only among individuals higher in effectance or social connection needs. In fact, for individuals low in those needs, anthropomorphism undermined intention.

Further, particular characteristics of anthropomorphized campaigns may vary in their success at eliciting compliance. In the original work, Ahn et al. noted that the promotion- versus prevention focus of the anthropomorphized message may be important. In a related vein, Rottman and colleagues note that harm-based appeals for environmental protection, such as those deployed in Ahn et al.’s Study 1 and our stimuli, can backfire [69]. Also, it may very well be the case that what is successful at eliciting compliance in environmental campaigns may need adaptation before deployment in other contexts (e.g., vaccination, voting, and second-hand smoking campaigns).

In short, anthropomorphism may be just one of many individual and message-framing influences on campaign compliance. As such, further research that delineates the necessary and sufficient cues and conditions for eliciting anthropomorphism-compliance effects will improve our understanding of when, how, and for whom adding anthropomorphizing cues will promote social cause compliance. More broadly, keeping in mind that the overall goal of social campaigns is to change behavior, we also wish to highlight the need for such research to not only measure intention, but to also assess behavioral compliance (see [70]), as Ahn et al. did in their Study 3.

## Conclusion

Across three experiments, we failed to observe the anthropomorphism-compliance effect: participants who viewed a poster advocating environmental behavior that contained anthropomorphic cues did not report higher intentions to comply than participants who viewed the same poster without anthropomorphic cues. We also failed to observe the previously documented effect of anthropomorphism on anticipated guilt, and found no effects on anticipated pride or anticipated regret. Paired with extant work on social cause anthropomorphism [1,3,67,68], we hope that these findings will inspire future research exploring the necessary and sufficient conditions under which anthropomorphic cues boost compliance with social causes. Such work will establish the boundary conditions of the anthropomorphism-compliance effect, which will be essential for the effective translation of research into practice.

## References

[1] Ahn H-K, Kim HJ, Aggarwal P (2014) Helping fellow beings: Anthropomorphized social causes and the role of anticipatory guilt. Psychol Sci 25: 224–229. 10.1177/0956797613496823 24192326

[2] Waytz A, Epley N, Cacioppo JT (2010) Social cognition unbound: Insights into anthropomorphism and dehumanization. Curr Dir Psychol Sci 19: 58–62. 10.1177/0963721409359302 24839358 PMC4020342

[3] Tam K-P, Lee S-L, Chao MM (2013) Saving Mr. Nature: Anthropomorphism enhances connectedness to and protectiveness toward nature. J Exp Soc Psychol 49: 514–521. 10.1016/j.jesp.2013.02.001

[4] Baumeister RF, Stillwell AM, Heatherton TF (1994) Guilt: An interpersonal approach. Psychol Bull 115: 243–267. 10.1037/0033-2909.115.2.243 8165271

[5] Basil DZ, Ridgway NM, Basil MD (2008) Guilt and giving: A process model of empathy and efficacy. Psychol Market 25: 1–23. 10.1002/mar.20200

[6] Basil DZ, Ridgway NM, Basil MD (2006) Guilt appeals: The mediating effect of responsibility. Psychol Market 23: 1035–1054. 10.1002/mar.20145

[7] Cole LM, Cohn ES, Rebellon CJ, Van Gundy KT (2013) Feeling guilty to remain innocent: The moderating effect of sex on guilt responses to rule-violating behavior in adolescent legal socialization. Psychol Crime Law 20: 722–740. 10.1080/1068316X.2013.854794

[8] Lindsey LLM (2005) Anticipated guilt as behavioral motivation. Hum Commun Res 31: 453–481. 10.1111/j.1468-2958.2005.tb00879.x

[9] Onwezen MC, Bartels J, Antonides G (2014) Environmentally friendly consumer choices: Cultural differences in the self-regulatory function of anticipated pride and guilt. J Environ Psychol 40: 239–248. 10.1016/j.jenvp.2014.07.003

[10] Peloza J, White K, Shang J (2013) Good and guilt-free: The role of self-accountability in influencing preferences for products with ethical attributes. J Mark 77: 104–119. 10.1509/jm.11.0454

[11] Nelissen RMA, Leliveld MC, van Dijk E, Zeelenberg M (2011) Fear and guilt in proposers: Using emotions to explain offers in ultimatum bargaining. Eur J Soc Psychol 41: 78–85. 10.1002/ejsp.735

[12] Stanger N, Kavussanu M, Ring C (2012) Put yourself in their boots: Effects of empathy on emotion and aggression. J Sport Exerc Psychol 34: 208–222. 22605362 10.1123/jsep.34.2.208

[13] Wang X (2011) The role of anticipated guilt in intentions to register as organ donors and to discuss organ donation with family. Health Commun 26: 683–690. 10.1080/10410236.2011.563350 22126126

[14] Steenhaut S, Van Kenhove P (2006) The mediating role of anticipated guilt in consumers’ ethical decision-making. J Bus Ethics 69: 269–288. 10.1007/s10551-006-9090-9

[15] Tangney JP, Stuewig J, Mashek DJ (2007) Moral emotions and moral behavior. Annu Rev Psychol 58: 345–372. 10.1146/annurev.psych.56.091103.070145 16953797 PMC3083636

[16] Elgaaied L (2012) Exploring the role of anticipated guilt on pro‐environmental behavior–a suggested typology of residents in France based on their recycling patterns. J Consum Mark 29: 369–377. 10.1108/07363761211247488

[17] Kaiser FG, Schultz PW, Berenguer J, Corral-Verdugo V, Tankha G (2008) Extending planned environmentalism: Anticipated guilt and embarrassment across cultures. Eur Psychol 13: 288–297. 10.1027/1016-9040.13.4.288

[18] Onwezen MC, Antonides G, Bartels J (2013) The norm activation model: An exploration of the functions of anticipated pride and guilt in environmental behaviour. J Econ Psychol 39: 141–153. 10.1016/j.joep.2013.07.005

[19] Epley N, Waytz A, Cacioppo JT (2007) On seeing human: A three-factor theory of anthropomorphism. Psychol Rev 114: 864–886. 10.1037/0033-295X.114.4.864 17907867

[20] Gray HM, Gray K, Wegner DM (2007) Dimensions of mind perception. Science 315: 619–619. 10.1126/science.1134475 17272713

[21] Gosselin P, Kirouac G, Doré FY (1995) Components and recognition of facial expression in the communication of emotion by actors. J Pers Soc Psychol 68: 83–96. 10.1037/0022-3514.68.1.83 7861316

[22] Provine RR, Krosnowski KA, Brocato NW (2009) Tearing: Breakthrough in human emotional signaling. Evol Psychol 7: 52–56.

[23] Stephan BCM, Breen N, Caine D (2006) The recognition of emotional expression in prosopagnosia: Decoding whole and part faces. J Inter Neuropsych Soc 12: 884–895. 10.1017/S1355617706061066 17064450

[24] Mascolo MF, Fischer KW (1995) Developmental transformations in appraisals for pride, shame, and guilt In: Tangney JP, Fischer KW, editors. Self-conscious emotions: The psychology of shame, guilt, embarrassment, and pride. New York: Guilford Press pp. 64–113.

[25] Tracy JL, Robins RW (2004) Putting the self into self-conscious emotions: A theoretical model. Psychol Inq 15: 103–125. 10.1207/s15327965pli1502_01

[26] Patrick VM, Chun HH, Macinnis DJ (2009) Affective forecasting and self-control: Why anticipating pride wins over anticipating shame in a self-regulation context. J Consum Psychol 19: 537–545. 10.1016/j.jcps.2009.05.006

[27] van der Schalk J, Bruder M, Manstead ASR (2012) Regulating emotion in the context of interpersonal decisions: The role of anticipated pride and regret. Front Psychol 3: 513 10.3389/fpsyg.2012.00513 23293615 PMC3536270

[28] Onwezen MC, Bartels J, Antonides G (2013) The self‐regulatory function of anticipated pride and guilt in a sustainable and healthy consumption context. Eur J Soc Psychol 44: 53–68. 10.1002/ejsp.1991

[29] Bell DE (1981) Explaining utility theory paradoxes by decision regret Organizations: multiple agents with multiple criteria. Berlin, Heidelberg: Springer Berlin Heidelberg pp. 28–39. 10.1007/978-3-642-45527-8_3

[30] Loomes G, Sugden R (1982) Regret theory: An alternative theory of rational choice under uncertainty. Econ J 92: 805–824. 10.2307/2232669

[31] Zeelenberg M (1999) Anticipated regret, expected feedback and behavioral decision making. J Behav Decis Mak 12: 93–106. 10.1002/(SICI)1099-0771(199906)12:2<93::AID-BDM311>3.0.CO;2-S

[32] Hetts JJ, Boninger DS, Armor DA, Gleicher F, Nathanson A (2000) The influence of anticipated counterfactual regret on behavior. Psychol Market 17: 345–368. 10.1002/(SICI)1520-6793(200004)17:4<345::AID-MAR5>3.0.CO;2-M

[33] Zeelenberg M, van Dijk WW, Van der Pligt J, Manstead ASR, van Empelen P, Reinderman D (1998) Emotional reactions to the outcomes of decisions: The role of counterfactual thought in the experience of regret and disappointment. Organ Behav Hum Decis Process 75: 117–141. 10.1006/obhd.1998.2784 9719660

[34] Koch EJ (2014) How does anticipated regret influence health and safety decisions? A literature review. Basic Appl Soc Psych 36: 397–412. 10.1080/01973533.2014.935379

[35] Rivis A, Sheeran P, Armitage CJ (2009) Expanding the affective and normative components of the theory of planned behavior: A meta-analysis of anticipated affect and moral norms. J Appl Soc Psychol 39: 2985–3019. 10.1111/j.1559-1816.2009.00558.x

[36] Sandberg T, Conner M (2010) Anticipated regret as an additional predictor in the theory of planned behaviour: A meta-analysis. Br J Soc Psychol 47: 589–606. 10.1348/014466607X258704 18039428

[37] Bagot KL, Masser BM, White KM (2015) Using an extended theory of planned behavior to predict a change in the type of blood product donated. Ann Behav Med. 10.1007/s12160-014-9677-9 25623894

[38] Godin G, Germain M, Conner M, Delage G, Sheeran P (2014) Promoting the return of lapsed blood donors: A seven-arm randomized controlled trial of the question-behavior effect. Health Psychol 33: 646–655. 10.1037/a0033505 23957902

[39] Masser BM, White KM, Hyde MK, Terry DJ, Robinson NG (2009) Predicting blood donation intentions and behavior among Australian blood donors: Testing an extended theory of planned behavior model. Transfusion 49: 320–329. 10.1111/j.1537-2995.2008.01981.x 19040598

[40] Nelissen RMA, van Someren DSI, Zeelenberg M (2009) Take it or leave it for something better? Responses to fair offers in ultimatum bargaining. J Exp Soc Psychol 45: 1227–1231. 10.1016/j.jesp.2009.06.004

[41] O'Carroll RE, Foster C, McGeechan G, Sandford K, Ferguson E (2011) The “ick” factor, anticipated regret, and willingness to become an organ donor. Health Psychol 30: 236–245. 10.1037/a0022379 21401258

[42] Kaiser FG (2006) A moral extension of the theory of planned behavior: Norms and anticipated feelings of regret in conservationism. Pers Individ Dif 41: 71–81. 10.1016/j.paid.2005.11.028

[43] Zeelenberg M, Breugelmans SM (2008) The role of interpersonal harm in distinguishing regret from guilt. Emotion 8: 589–596. 10.1037/a0012894 18837609

[44] Keith LT, Tornatzky LG, Pettigrew LE (1974) An analysis of verbal and nonverbal classroom teaching behaviors. J Exp Educ 42: 30–38. 10.1080/00220973.1974.11011490

[45] Rosenfeld HM (1967) Nonverbal reciprocation of approval: An experimental analysis. J Exp Soc Psychol 3: 102–111. 10.1016/0022-1031(67)90040-6

[46] Schultz PW, Nolan JM, Cialdini RB, Goldstein NJ, Griskevicius V (2007) The constructive, destructive, and reconstructive power of social norms. Psychol Sci 18: 429–434. 10.1111/j.1467-9280.2007.01917.x 17576283

[47] Rosenfeld HM (1966) Approval-seeking and approval-inducing functions of verbal and nonverbal responses in the dyad. J Pers Soc Psychol 4: 597–605. 10.1037/h0023996

[48] Stevens C, Sidener TM, Reeve SA, Sidener DW (2011) Effects of behavior-specific and general praise, on acquisition of tacts in children with pervasive developmental disorders. Res Autism Spectr Disord 5: 666–669. 10.1016/j.rasd.2010.08.003

[49] Swim JK, Bloodhart B (2013) Admonishment and praise: Interpersonal mechanisms for promoting proenvironmental behavior. Ecopsychology 5: 24–35. 10.1089/eco.2012.0065

[50] Webster JM, Duvall J, Gaines LM, Smith RH (2003) The roles of praise and social comparison information in the experience of pride. J Soc Psychol 143: 209–232. 10.1080/00224540309598441 12735519

[51] Williams LA, DeSteno D (2008) Pride and perseverance: The motivational role of pride. J Pers Soc Psychol 94: 1007–1017. 10.1037/0022-3514.94.6.1007 18505314

[52] Williams LA, DeSteno D (2009) Pride: Adaptive social emotion or seventh sin? Psychol Sci 20: 284–288. 10.1111/j.1467-9280.2009.02292.x 19207690

[53] Perrin JL (2011) Emotional responses to environmental messages and future behavioral intentions. Appl Environ Educ Commun 10: 146–157. 10.1080/1533015X.2011.603612

[54] Searles K (2010) Feeling good and doing good for the environment: The use of emotional appeals in pro-environmental public service announcements. Appl Environ Educ Commun 9: 173–184. 10.1080/1533015X.2010.510025

[55] Meijnders AL, Midden CJ, Wilke HA (2001) Communications about environmental risks and risk‐reducing behavior: The impact of fear on information processing. J Appl Soc Psychol 31: 754–777. 10.1111/j.1559-1816.2001.tb01412.x

[56] Faul F, Erdfelder E, Lang A-G, Buchner A (2007) G* Power 3: A flexible statistical power analysis program for the social, behavioral, and biomedical sciences. Behav Res Methods 39: 175–191. 10.3758/BF03193146 17695343

[57] Smithson M (2003) Confidence intervals Thousand Oaks: Sage Publications.

[58] Wuensch KL (2012) Using SPSS to obtain a confidence interval for Cohen’s d. Retrieved February 15, 2015, from http://coreecuedu/psyc/wuenschk/SPSS/CI-d-SPSSpdf.

[59] Funder DC, Levine JM, Mackie DM, Morf CC, Sansone C, Vazire S, et al (2014) Improving the dependability of research in personality and social psychology. Pers Soc Psychol Bull 18: 3–12. 10.1177/1088868313507536 24214149

[60] Wilson DB (2006) Meta-analysis macros for SAS, SPSS, and Stata. Retrieved February 1, 2015, from http://mason.gmu.edu/~dwilsonb/ma.html.

[61] Lipsey MW, Wilson DB (2000) Practical meta-analysis Sage Publications.

[62] Bateson M, Callow L, Holmes JR, Roche MLR, Nettle D (2013) Do images of “watching eyes” induce behaviour that is more pro-social or more normative? A field experiment on littering. PLoS One 8: e82055 10.1371/journal.pone.0082055 24339990 PMC3855385

[63] Powell KL, Roberts G, Nettle D (2012) Eye images increase charitable donations: Evidence from an opportunistic field experiment in a supermarket. Ethology 118: 1096–1101. 10.1111/eth.12011

[64] Ekström M (2011) Do watching eyes affect charitable giving? Evidence from a field experiment. Exp Econ 15: 530–546. 10.1007/s10683-011-9312-6

[65] Ernest-Jones M, Nettle D, Bateson M (2011) Effects of eye images on everyday cooperative behavior: A field experiment. Evol Hum Behav 32: 172–178. 10.1016/j.evolhumbehav.2010.10.006

[66] Francey D, Bergmüller R (2012) Images of eyes enhance investments in a real-life public good. PLoS One 7: e37397 10.1371/journal.pone.0037397 22624026 PMC3356250

[67] Tam K-P (2014) Anthropomorphism of nature and efficacy in coping with the environmental crisis. Soc Cogn 32: 276–296. 10.1521/soco.2014.32.3.276

[68] Tam K-P (2015) Are anthropomorphic persuasive appeals effective? The role of the recipient's motivations. Br J Soc Psychol 54: 187–200. 10.1111/bjso.12076 24898876

[69] Rottman J, Kelemen D, Young L (2015) Hindering harm and preserving purity: How can moral psychology save the planet. Philos Compass 10: 134–144. 10.1111/phc3.12195

[70] Baumeister RF, Vohs KD, Funder DC (2007) Psychology as the science of self-reports and finger movements: Whatever happened to actual behavior? Perspect Psychol Sci 2: 396–403. 10.1111/j.1745-6916.2007.00051.x 26151975

